# Low-Frequency Bandgap Characterization of a Locally Resonant Pentagonal Phononic Crystal Beam Structure

**DOI:** 10.3390/ma17071702

**Published:** 2024-04-08

**Authors:** Shengke Zhang, Denghui Qian, Zhiwen Zhang, Haoran Ge

**Affiliations:** School of Naval Architecture & Ocean Engineering, Jiangsu University of Science and Technology, Zhenjiang 212100, China

**Keywords:** phononic crystal double-layer beam, local resonance, energy band structure, transmission attenuation, vibration modes, displacement field

## Abstract

This paper proposes a local resonance-type pentagonal phononic crystal beam structure for practical engineering applications to achieve better vibration and noise reduction. The energy band, transmission curve, and displacement field corresponding to the vibration modes of the structure are calculated based on the finite element method and Bloch-Floquet theorem. Furthermore, an analysis is conducted to understand the mechanism behind the generation of bandgaps. The numerical analysis indicates that the pentagonal unit oscillator creates a low-frequency bandgap between 60–70 Hz and 107–130 Hz. Additionally, the pentagonal phononic crystal double-layer beam structure exhibits excellent vibration damping, whereas the single-layer beam has poor vibration damping. The article comparatively analyzes the effects of different parameters on the bandgap range and transmission loss of a pentagonal phononic crystal beam. For instance, increasing the thickness of the lead layer leads to an increase in the width of the bandgap. Similarly, increasing the thickness of the rubber layer, intermediate plate, and total thickness of the phononic crystals results in a bandgap at lower frequencies. By adjusting the parameters, the beam can be optimized for practical engineering purposes.

## 1. Introduction

The economic and technological development of human society has led to the creation and use of various mechanical equipment, providing mankind with a convenient way of life. However, the resulting noise problem has been a cause for concern. Noise not only reduces the service life of a machine and affects its precision, but it also poses a risk to people’s hearing and can lead to fatal diseases, including cancer. Therefore, it is crucial to reduce vibration and noise for the sake of people’s health. To mitigate the impact of noise on the environment and people’s lives, there are three primary methods of noise control: the direct suppression of the noise source, the control of the noise propagation path, and the protection of the receiver. Controlling the propagation pathway is a common method for reducing the noise pollution caused by engineering and mechanical equipment. This can be achieved by using soundproof walls to block the spread of noise. Directly inhibiting the sound source is often difficult. Scholars have been struggling to determine which materials or structures of soundproofing mediums are most effective in blocking noise. In recent years, scholars both domestically and internationally have conducted extensive research on phononic crystals. It was found that elastic waves are significantly suppressed when propagating through materials and structures with periodic distributions of elastic constants and densities due to their periodic structure. The emergence of this structure offers new solutions for problems such as vibration and noise reduction. This is expected to lead to breakthroughs in this field in the future.

Phononic crystals [1,2,3] are materials or structures that have a periodic distribution of elastic constants and densities. Phononic crystals suppress or prohibit elastic wave propagation in certain frequency ranges due to their periodic structure. This makes them a promising solution for vibration and noise reduction. In recent years, scientists have applied phononic crystals in the fields of marine hydroacoustic detection [4], automobiles [5], and architecture. These crystals have shown potential for various applications due to their unique properties. Two mechanisms generate bandgaps in phononic crystals: Bragg scattering [6,7] and local resonance [8,9,10,11]. The first phenomenon is primarily caused by the periodicity of the structure, while the second is mainly due to the resonance properties of the individual crystal cells. The Bragg scattering principle [12] states that elastic waves propagating in phononic crystals with a periodic structure experience the formation of a unique dispersion relation, or energy band structure. The frequency range between the dispersion relation curves is known as the bandgap. The principle of local resonance states that a strong coupling between an elastic wave and a crystal cell structure occurs when their frequencies are close, resulting in the emergence of a bandgap. Research has shown that locally resonant phononic crystals [13,14,15] contain oscillators as a result of a softer composite block in their middle, which connects the harder plate to the substrate. When the frequency of the elastic wave coincides with the resonance frequency of the oscillator, a very strong coupling occurs between the oscillator and the elastic wave. This coupling can inhibit or even prohibit the propagation of the elastic wave, resulting in the formation of a bandgap. Due to the low-frequency characteristics of local resonance-type phononic crystals, scholars have shown increased interest in using them to achieve low-frequency vibration and noise reduction.

In their study, Khales et al. [16] examined a phononic crystal beam structure composed of a linear lattice array of square pillars on a beam. They demonstrated the existence of a fully ultrasonic bandgap in this structure both theoretically and experimentally. Additionally, they predicted that this structure would reduce energy loss in machinery operating in a high-frequency range. Shen et al. [17] proposed a low-frequency vibrational energy generator that utilizes a locally resonant phononic crystal plate. The local resonance at the flat-band frequency of the phononic crystal is verified to result in a spiral beam with a significant deformation perpendicular to the plate when using the finite element method. The experimental design enables the phonon crystal plate structure to collect widely distributed vibration energy. Zhou et al. [18] experimentally and numerically confirmed the low-frequency vibration isolation capabilities of a new hybrid phononic crystal plate. The structure proposed comprises of two periodic double-sided composite resonators deposited on a two-component plate. The finite element method was used to calculate the eigenmodes, dispersion relations, transmission spectra, and displacement fields. Vibration tests were conducted on the prepared specimens to confirm the transmission loss of elastic waves within a specific frequency range. The effectiveness of the proposed structure as an alternative for broadband low-frequency vibration isolation was demonstrated. Kuang et al. [19] conducted a numerical investigation of the phonon energy band structure of two-dimensional solid phononic crystals. The crystals consisted of lattices with different symmetries and scatterers (hexagon, circle, square, and triangle) with varying shapes (triangular, hexagonal, and square), orientations, and sizes. The study provides insights for designing beam-and-plate-type structures for phononic crystals. Li et al. [5] proposed a new type of phononic crystal plate based on common two-dimensional convex phononic crystals combined with micro-perforated plates, and applied this new combined phononic crystal structure to the front panel of an automobile, which had a significant noise reduction effect on the driver’s cab. Xiao et al. [20] designed a plate resonator using a periodic arrangement of phononic crystal beams supported by phononic crystals through analysis and experimentation with local resonance-type phononic crystals. The experimental results indicate that the complete bandgap ranges from 465 Hz to 860 Hz. This suggests that the acoustic sub-panel has potential applications in low-frequency mechanical vibration attenuation and low-frequency audible sound insulation. Liu et al. [21] proposed a two-dimensional phononic crystal model based on the T-square fractal for a comprehensive study of Bragg scattering and locally resonant fractal phononic crystals. The energy band structure between fractal and non-fractal phononic crystals at the same filling ratio differs significantly when using the finite element method. The fractal design significantly affects the energy band structure of two-dimensional phononic crystals. Yin et al. [22] proposed a novel structure for a compression–torsion coupled phononic crystal beam, which includes a curved beam. They optimized this structure using a genetic algorithm. This work opens up new possibilities for designing phononic crystal beam structures.

In recent years, several structural forms of phononic crystals have gained attention. However, only a limited number of phononic crystals have been studied in the frequency range below 200 Hz, where the bandgap and transmission attenuation bands are located. Among these structures, the study of phononic crystal beams [12,23] and phononic crystal plates [24,25,26] is particularly important for practical engineering applications. The development of phononic crystals in beam and plate form, made of lead, steel, and rubber, has contributed to both the field of engineering and the quality of life by reducing vibration and noise at frequencies below 200 Hz. This paper presents a novel localized resonant pentagonal phononic crystal. First, the author builds a pentagonal unit oscillator and takes five pentagonal unit oscillators to form a union by arranging them periodically in the vertical direction. The upper and lower surfaces of this structure are affixed with two thin metal plates to form the cell structure. Then, five cell structures are arranged laterally and periodically to form a two-layer beam structure of a phonon crystal. The bandgap characteristics of phonon crystal beams were studied using the finite element method with COMSOL Multiphysics 6.1. The article analyzes the mechanism of bandgap formation by comparing the band structure and transmission loss of a phonon crystal single-layer beam and double-layer beam. It also studies the influence of different parameters on bandgap and transmission loss to optimize the structural model for practical engineering applications.

## 2. Model and Methods

**Figure 1:**
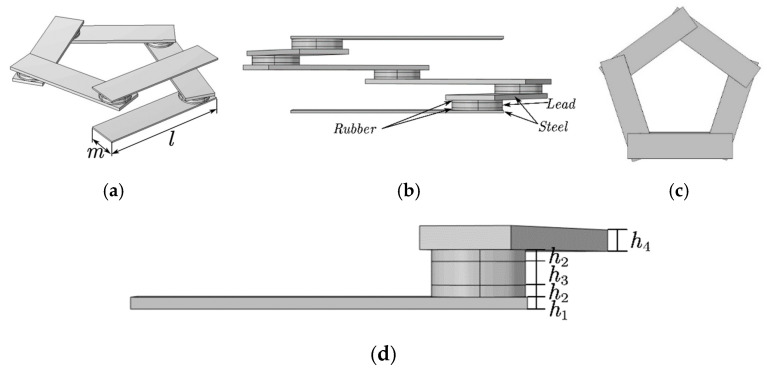
Pentagonal unit cell structure. (**a**) Panorama. (**b**) Main view. (**c**) Top view. (**d**) Partial unit cell structure.

**Figure 2:**
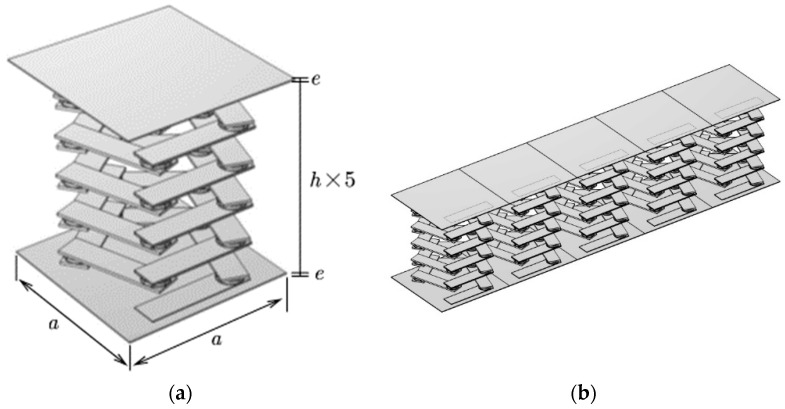
Modeling of the double-layer beam structure of phononic crystals. (**a**) Cell structure of phononic crystal beams. (**b**) Phononic crystal beam structure.

Based on the elastic constants and periodic structure of the phononic crystal, a pentagonal unit oscillator structure is established, as shown in Figure 1. It can be seen from Figure 1a that the pentagonal element oscillator is composed of 6 steel plates and 5 composite blocks attached at a certain angle. The top and bottom are the two thinner steel plates, while the middle is the four thicker steel plates. The composite block is composed of a rubber–lead block and rubber interleaving, and the periodic structure distribution forms the vibrator. The cell structure is formed by attaching two plates above and below it, as shown in Figure 2a. The formed cell is arranged transversely and periodically to form a phononic crystal bilayer beam structure, as shown in Figure 2b. In the structural model, the lattice constants, the thicknesses of the upper and lower plates of the cell structure, the thicknesses of the upper and lower plates of the pentagonal unit oscillator, the thickness of the rubber layer, the thickness of the lead layer, the thicknesses of the four plates in the middle of the pentagonal unit oscillator, and the total height of the pentagonal unit oscillator are a, e, $h_{1}$, $h_{2}$, $h_{3}$, $h_{4}$, and h, respectively, as shown in Figure 1d. The length and width of one plate in the pentagonal unit oscillator are l and m, respectively, as shown in Figure 1a.

The elastic fluctuation equation in a perfectly linearly elastic, isotropic medium independent of external forces is

(1) $$-\rho \omega^{2}u(r)=(\lambda +\mu )\nabla (\nabla \cdot u(r))+\mu \nabla^{2}u(r)$$

In the above equation, r is the position vector, ∇ is the Hamiltonian operator, u is the particle displacement vector, ρ is the density of the medium, μ and λ are the Lame constant of the medium, and ω is the characteristic circular frequency.

The energy band structure of a locally resonant pentagonal phononic crystal beam was calculated using the finite element method in COMSOL Multiphysics 6.1. The region is discretized by dividing the finite element mesh into tetrahedral meshes. For the boundary conditions, the pressure-free free boundary condition is used for all non-contact surfaces of the pentagonal phononic crystal, and the Bloch–Floquet periodic boundary condition is used for the contact surfaces of the neighboring unitary oscillators, viz:

(2) $$u(r+a)=u(r)e^{i(k\cdot a)}$$

In the above equation a is the lattice constant. The setup of the periodic boundary conditions for the different structures is shown below.

**Figure 3:**
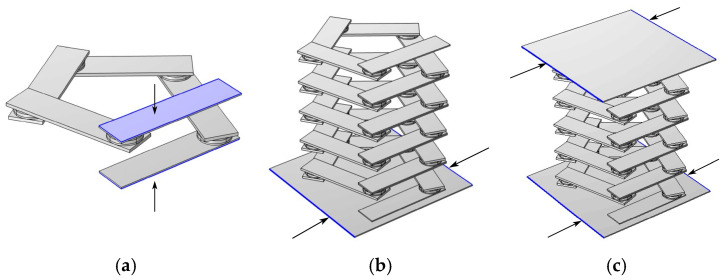
Set periodic boundary conditions on the surface indicated by the arrow. (**a**) Setting of periodic boundary conditions for unit oscillators. (**b**) The setting of periodic boundary conditions for single-layer phononic crystal beams. (**c**) The setting of periodic boundary conditions for phononic crystal bilayer beams.

Substituting the periodic boundary condition Equation (2) into the finite element characteristic equation for free vibration yields

(3) $$Ku=\omega^{2}Mu$$

where K is the stiffness matrix and M is the mass matrix, both of which contain Bloch wavevector coupling terms. For the unitary oscillator, as shown in Figure 3a, the two boundaries in the z-direction are taken as the source terms, the periodic boundary conditions are used for the upper and lower boundaries, and the wavevector kz is added in the z-direction, and the wavevector is scanned parametrically. The scan range, frequency and step size are set as desired, so the wavevector traverses the first irreducible Brillouin to obtain the energy band structure. For each given value of kz, the corresponding eigenfrequency is obtained by solving for the eigenvalue. The energy band diagram shows a wider bandgap between the dispersion curves, which is in a lower frequency range. This low-frequency bandgap has broad potential for practical engineering applications. It can effectively suppress or prohibit noise propagation within this frequency range. To be applicable in real-life scenarios, a single pentagonal oscillator is insufficient. Therefore, a pentagonal phonon crystal beam cell structure, as depicted in Figure 2a, must be constructed. The phonon crystal beam cell structure can be used in practical engineering by arranging it transversely and periodically to form a phonon crystal beam structure. This structure has potential applications in various fields due to its unique properties.

The study demonstrates that the transmission loss of the finite-period phonon crystal structure and the wireless-period phonon crystal structure coincide. Therefore, the finite-period phonon crystal structure can be used in place of the infinite-period phonon crystal structure for this study. In practice, it is not possible to create a phononic crystal structure with an infinite period. Therefore, the best possible vibration and noise reduction can only be achieved within a limited range. In this paper, the author establishes the phonon crystal double-layer beam structure formed by the lateral periodic arrangement of five phonon crystal beam cells, as shown in Figure 2b.The sample utilizes the finite element method with pressure-free free boundary conditions for the non-contact surfaces and Bloch–Floquet periodic boundary conditions for the contact surfaces, as shown in Figure 3c, to derive the energy band diagrams of the pentagonal phononic crystal bilayer beam structure.

## 3. Numerical Results and Analyses

### 3.1. Energy Band Structure and Transmission Curves of Pentagonal Unit Oscillators

**Figure 4:**
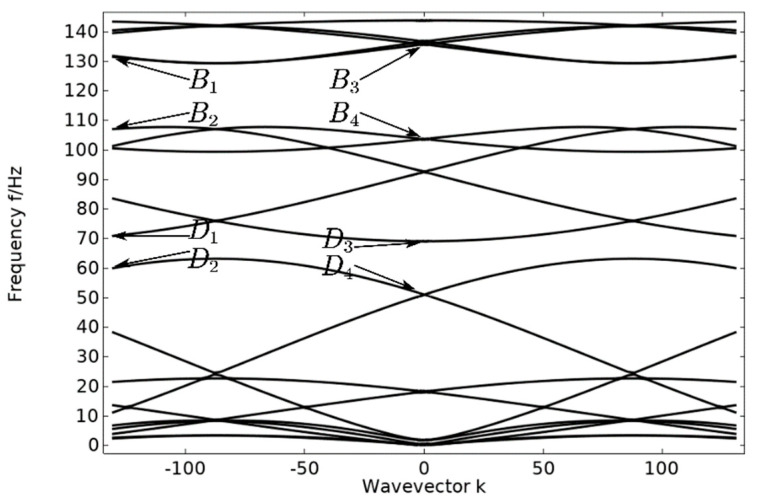
Energy band diagram of a pentagonal unit oscillator.

The energy band structure of the pentagonal unit oscillator was calculated using the finite element method with the assistance of COMSOL Multiphysics 6.1, as depicted in Figure 4. The material and geometrical parameters utilized in the calculation are presented in Table 1 and Table 2. In order to verify the correctness of the energy band structure, five unit cell oscillators are periodically arranged in the vertical direction to form a crystal cell structure and their transmission curves are calculated. Verify the correctness of bandgap occurrence by comparing the energy band diagram and the transmission curve diagram.

**Figure 5:**
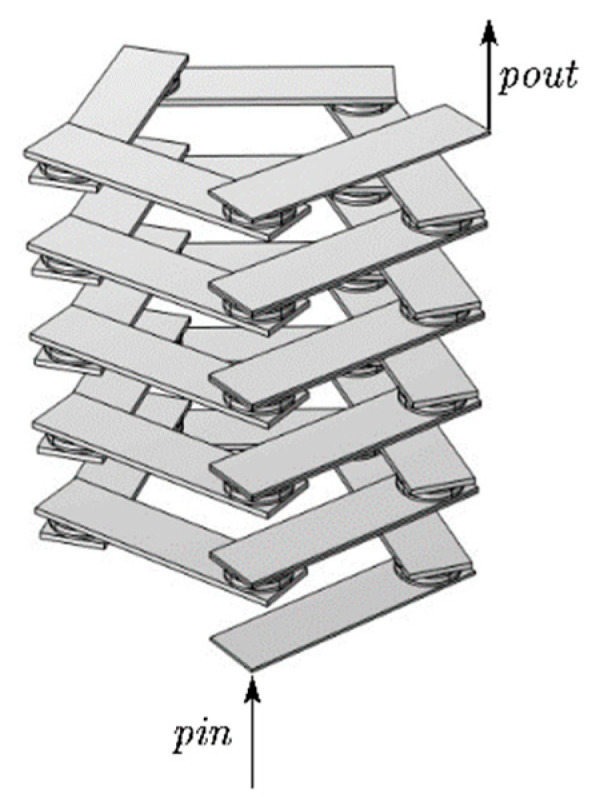
Cell structure formed by vertical arrangement of five unit cell oscillators.

**Figure 6:**
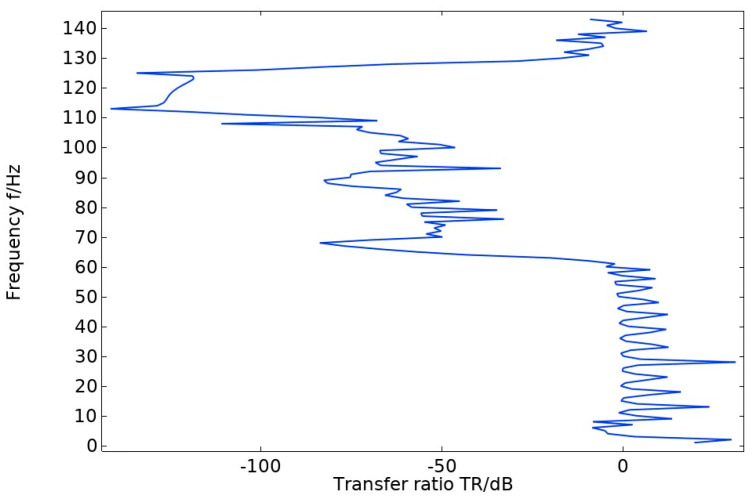
Transmission profile of pentagonal oscillator cell structure.

The energy band diagram in Figure 4 shows that the bandgap is more pronounced in two frequency ranges: 60–70 Hz and 107–130 Hz, with a total width of 33 Hz. The bandgap frequency is significantly low, making it important for studying the vibration and noise reduction of phononic crystals at low frequencies. After arranging the unit oscillator vertically and periodically to form a crystal cell, the excitation and response points are added to the lower and upper plates of the cell structure, as shown in Figure 5. The transmission curve obtained by scanning the energy band graph with COMSOL Multiphysics 6.1 in the frequency domain of 0–143 Hz is shown in Figure 6. The transmission curve shows that the frequency band of transmission attenuation corresponds well with the frequency band of the bandgap range, which confirms the occurrence of the bandgap. To investigate the reason for the appearance of the bandgap, we analyze the displacement field corresponding to the eigenmode at the critical point of the bandgap.

**Figure 7:**
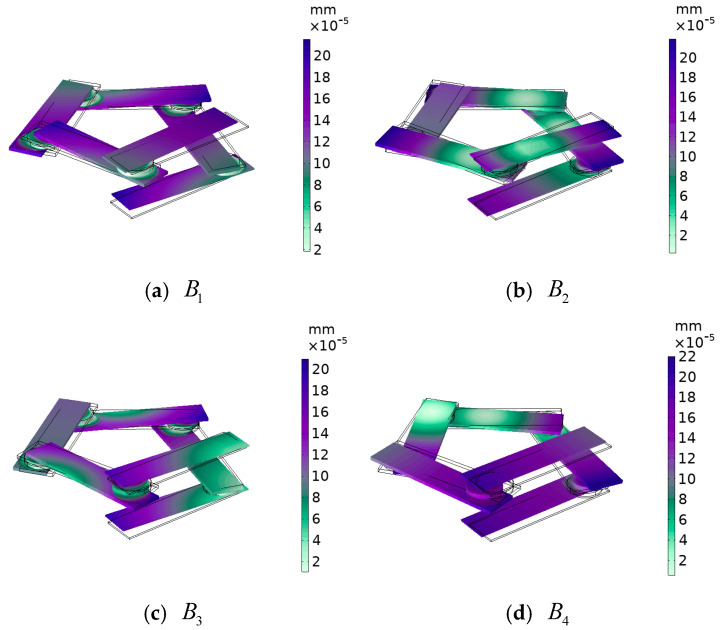
The displacement field corresponding to the annotated modes in the high frequency range of Figure 4.

In the pentagonal unit vibration energy band structure, the displacement fields corresponding to vibration modes $B_{1}$ and $B_{3}$, as shown in Figure 7, are mainly due to the horizontal rotational vibration of the steel plate caused by the shear vibration of the composite block (rubber–lead–rubber) in the xoy plane. However, since each steel plate generates a horizontal rotational vibration, the unit oscillator still maintains the pentagonal structure and undergoes small changes, and the horizontal rotational vibration of each steel plate is coupled with each other to achieve dynamic equilibrium. For modes $B_{2}$ and $B_{4}$, the steel plate is mainly subjected to shear vibration in the xoy plane, at which time the frequency of the elastic wave is similar to the resonance frequency of the composite block, and the elastic wave strongly couples with the phononic crystal, so that a band of frequencies ranging from 107 Hz to 130 Hz is opened. The reason for the horizontal shear vibration of the steel plate may be that the pentagonal element vibrator is at the critical point of band truncation, and the bandgap just begins to appear.

**Figure 8:**
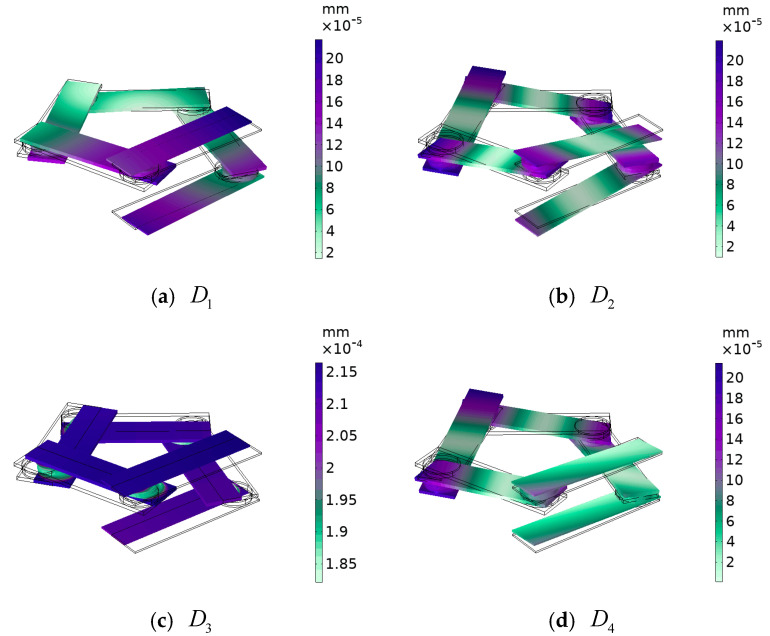
The displacement field corresponding to the annotated mode in the low-frequency range shown in Figure 4.

For vibrational modes $D_{1}$ and $D_{3}$, as shown in Figure 8, which are similar to vibrational modes $B_{1}$ and $B_{3}$ above, vibrational modes $D_{2}$ and $D_{4}$ are similar to $B_{2}$ and $B_{4}$. The difference is that this local resonance of the pentagonal phononic crystal oscillator only opens the bandgap between 60 Hz and 70 Hz, but provides a basis for the subsequent opening of a wider bandgap. And with such a low-frequency bandgap, there is good potential for practical applications.

### 3.2. The Band Structure and Transmission Curve of a Monolayer Beam of Pentagonal Phononic Crystals

**Figure 9:**
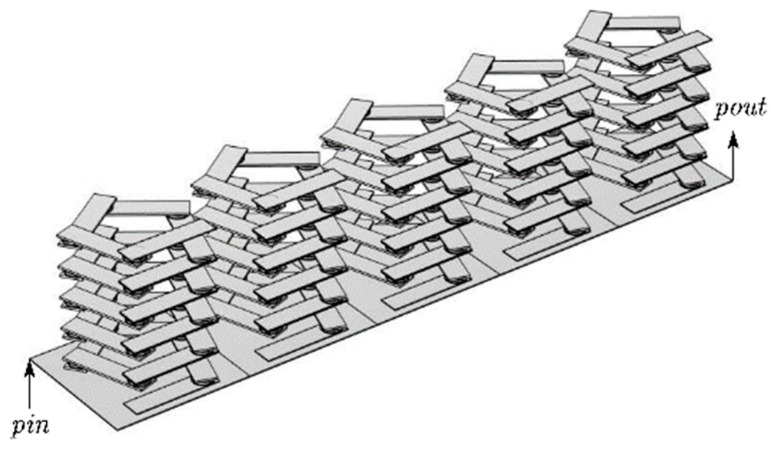
A single-layer beam structure of pentagonal phononic crystals.

**Figure 10:**
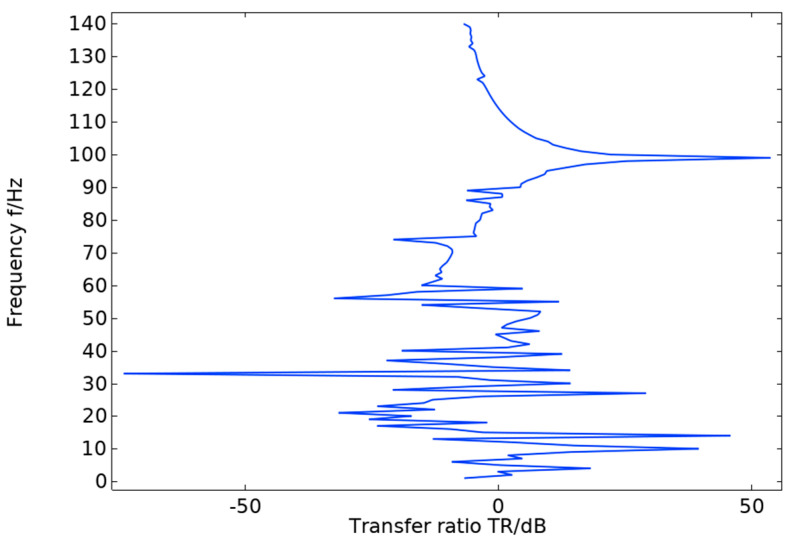
Transmission curve diagram of the single-layer beam.

**Figure 11:**
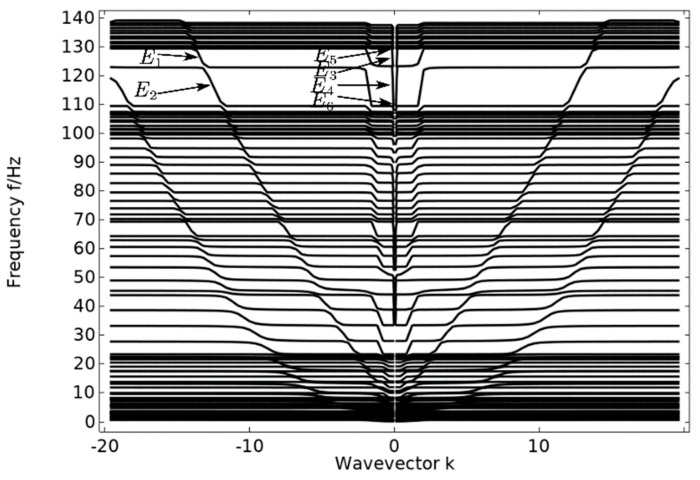
Energy band diagram of the single-layer beam.

A steel plate is attached to the lower surface of the cell formed by the vertical periodic arrangement of unit oscillators to form a new cell structure. Then, the new cell structure is arranged laterally and periodically to form a single layer beam structure of pentagonal phonon crystals. For the pentagonal phononic crystal single-layer beam structure, the excitation and response points are added on both sides of the lower plate, as shown in Figure 9, to obtain its transmission profile, as shown in Figure 10. From the transmission curve, it can be observed that there is no significant frequency band of vibration attenuation in the single-layer beam. In order to investigate the cause, the vibration mode marked in Figure 11 is selected for analysis.

**Figure 12:**
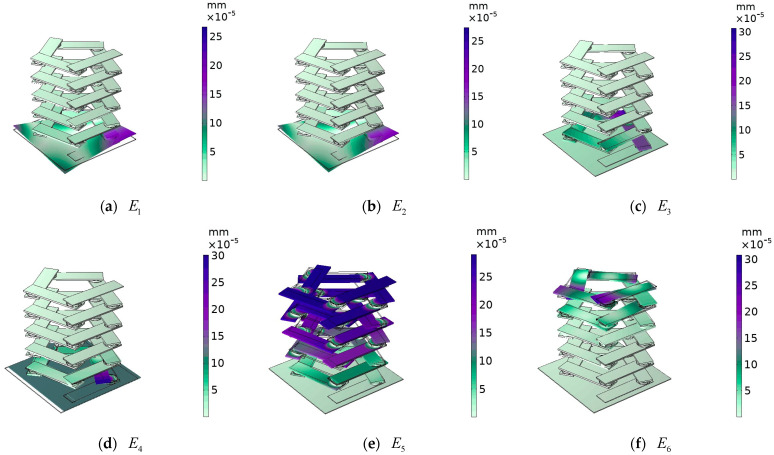
The displacement field corresponding to the labeled modes in Figure 9.

In the single-layer beam cell energy band structure, the displacement fields corresponding to vibration modes $E_{1}$ and $E_{2}$, as shown in Figure 12, are both longitudinal torsional vibrations of the lower steel plate. There is a steel plate in which one pair of corners vibrates longitudinally upward in torsion and the other pair of corners vibrates longitudinally downward in torsion. This corresponds to the antisymmetric vibration of the corners of the adjacent edges and the symmetric vibration of the corners of the opposite edges, so that the vibration phase of the lower panel reaches a longitudinal dynamic equilibrium. For vibration mode $E_{3}$, an oscillator at the lowest level vibrates rotationally in the xoy plane, and in vibration mode $E_{4}$, the lower steel plate vibrates horizontally in the xoy plane. Vibration modes $E_{3}$ and $E_{4}$ are the vibration of the oscillator and plate on the lower side in the xoy plane, and these vibrations are coupled with each other to achieve lateral vibration dynamic equilibrium. For vibrational modes $E_{5}$ and $E_{6}$, both are at the position of the critical point of the bandgap range, vibrational mode $E_{6}$ is the position of the beginning of the local resonance, where the uppermost oscillators start to vibrate, while vibrational mode $E_{5}$ is at the critical point of the end of the local resonance, where the majority of the oscillators have already started to produce strong vibrations. From the above analysis, it is concluded that even though local resonance can occur in the cellular structure of a single-layer beam, when both the excitation point and the response point are located on the lower side of the single-layer beam, the oscillator cannot function, the elastic wave can still be propagated in the lower side plate, and the elastic wave does not have any significant transmission loss. This shows that it is not always possible to achieve vibration and noise reduction using a phononic crystal oscillator, and that the effects of a number of factors must often be considered.

### 3.3. Band Structure and Transport Curve of Pentagonal Phonon Crystal Double-Layer Beams

**Figure 13:**
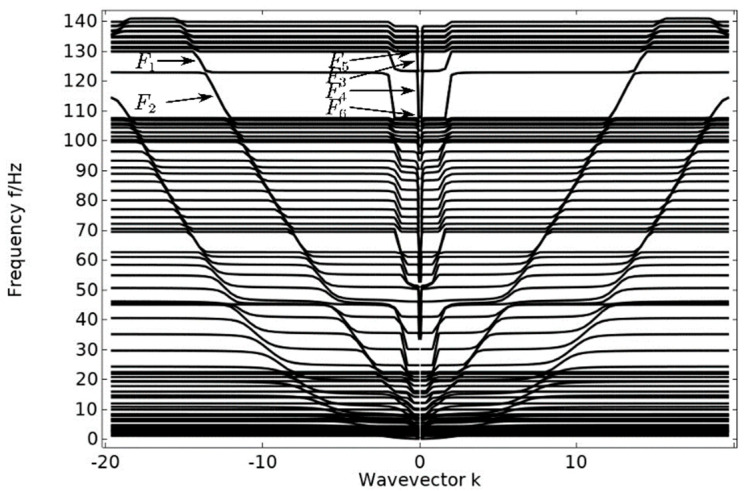
Band diagram of a double beam.

**Figure 14:**
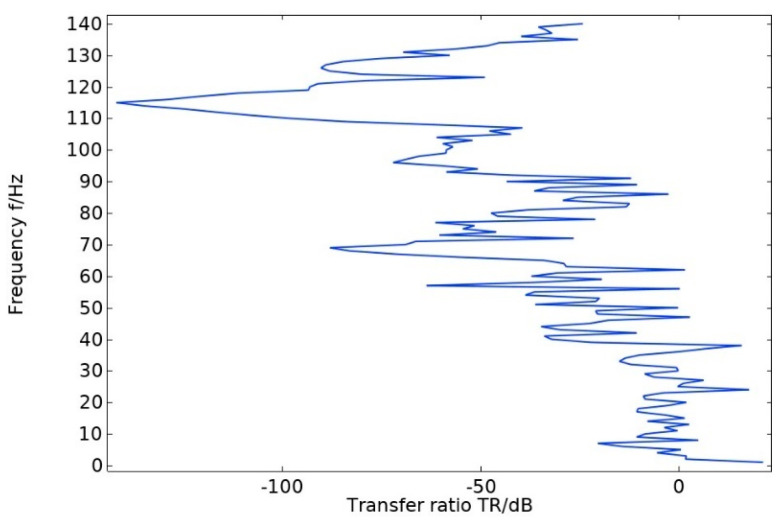
Transmission curve of double beam.

A steel plate is attached to both the upper and lower surfaces of the unitary oscillator cell structure to form a pentagonal double-layer beam cell structure, to which the cell structure is periodically arranged transversely to form a pentagonal phononic crystal double-layer beam structure. As can be seen from the band diagram of the double-layer beam in Figure 13, there is no obvious bandgap, but the transmission curve diagram in Figure 14 shows an obvious transmission attenuation band. Therefore, the vibration modes of the points marked in the band diagram are selected for analysis, and the causes of the transmission attenuation frequency bands are studied.

**Figure 15:**
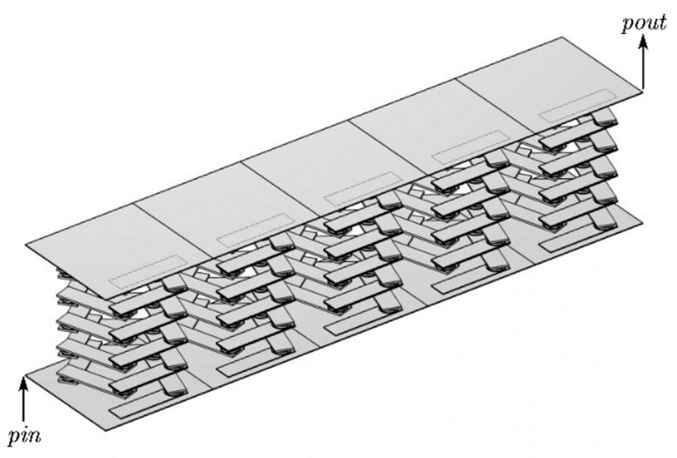
Pentagonal phonon crystal double-beam structure.

**Figure 16:**
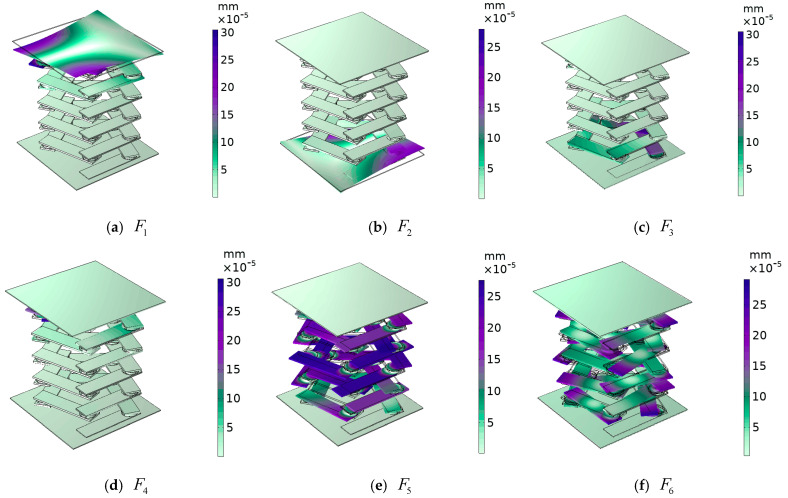
The displacement field corresponding to the labeled modes in the figure.

The excitation and response points of the pentagonal phononic crystal double-layer beam structure are shown in Figure 15. In the energy band structure of a double-layer beam, the displacement fields corresponding to vibration modes $F_{1}$ and $F_{2}$, as shown in Figure 16, are torsional vibrations of the upper (lower) plate in the longitudinal direction. In vibration mode $F_{1}$, the upper plate torsionally vibrates in the longitudinal direction, while the lower side plate and the intermediate vibrator do not vibrate. In vibration mode $F_{2}$, the lower plate vibrates in the longitudinal direction in torsion, and the intermediate vibrator and the upper side plate do not vibrate. Therefore, the torsional vibration of the lower side plate of vibration mode $F_{2}$ is symmetrical with the torsional vibration of the upper side plate of vibration mode $F_{1}$, so as to achieve the dynamic equilibrium of vibration in the longitudinal direction. In vibration modes $F_{3}$ and $F_{4}$, the corresponding displacement fields are such that the uppermost (lowermost) vibrator vibrates and neither the upper nor lower plates vibrate, and the elastic waves in the frequency range are well suppressed. Vibration modes $F_{5}$ and $F_{6}$ are the displacement fields corresponding to the critical points of the elastic wave attenuation band. Vibration mode $F_{6}$ is the vibration mode at the beginning of the appearance of the attenuation band, where the composite block of each oscillator in the middle rotates and vibrates in the xoy plane, while the upper and lower plates do not vibrate. This is due to the fact that at the beginning of the elastic wave transmission decay, the vibration frequency of the composite block (rubber–lead–rubber) inside the phononic crystal is close to the elastic wave frequency, and the composite block generates a local resonance. The elastic wave is localized in the composite block and the energy of the elastic wave is also concentrated in the composite block, resulting in the elastic wave not being able to propagate further and the transmission being suppressed, resulting in a band with increased transmission loss. Vibration mode $F_{5}$ is the termination point of the transmission decay band, and the vibration of the double-layer beam cell structure of the pentagonal phononic crystal also occurs only in the middle oscillator, while the upper and lower plates do not vibrate. The mechanism is similar to that of the onset vibration mode $F_{6}$ that occurs in the transmission-fading band. However, the rotational and horizontal vibrations of the intermediate oscillator in the xoy plane are more pronounced in the displacement field corresponding to vibrational mode $F_{5}$. This is due to the fact that the $F_{5}$ vibration mode is already at the end of the vibration-damping band, where the vibration amplitude increases significantly and the transmission loss decreases. No matter which vibration mode corresponds to the displacement field, only one side of the plate can produce vibration at most, and the other side of the plate does not produce vibration, indicating that the propagation of elastic waves is suppressed. The above analysis of the vibration modes of the points marked in the energy band diagram of the double-layer beam in Figure 13 is an effective proof of the emergence of the elastic wave vibration-damping band.

To better explain why there is no significant bandgap in the energy band diagram and why there is a significant vibrational attenuation band in the transmission curve diagram, the vibration modes of double-layer beams falling in the non-transmission attenuation bands with frequencies of 5 Hz, 17 Hz, and 30 Hz and the vibration modes of double-layer beams falling in the transmission attenuation bands with frequencies of 67 Hz, 110 Hz, and 120 Hz are selected.

**Figure 17:**
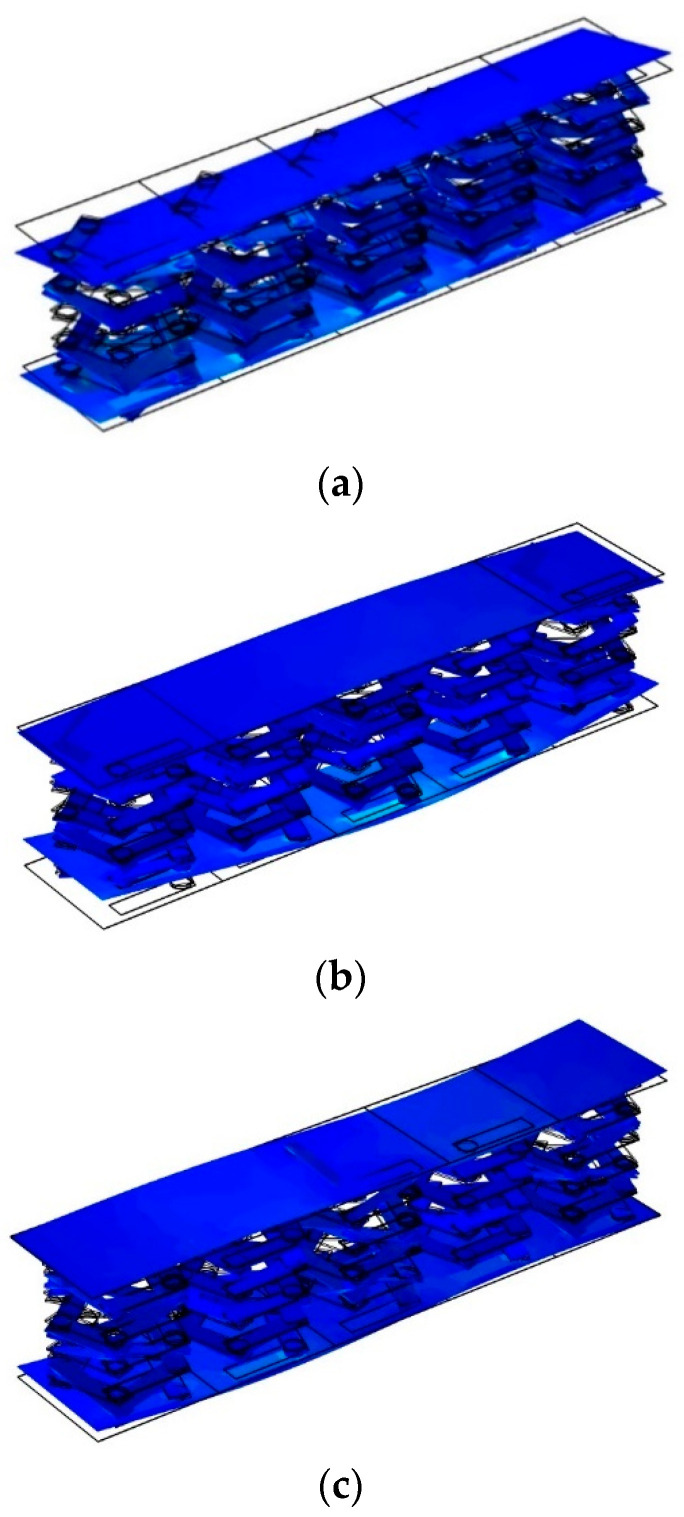
Vibration modes falling in the non-transmission attenuation frequency band. (**a**) A vibration mode with a frequency of 5 Hz falling in the non-transmission attenuation frequency band. (**b**) A vibration mode with a frequency of 5 Hz falling in the non-transmission attenuation frequency band. (**c**) A vibration mode with a frequency of 17 Hz falling in the non-transmission attenuation frequency band.

**Figure 18:**
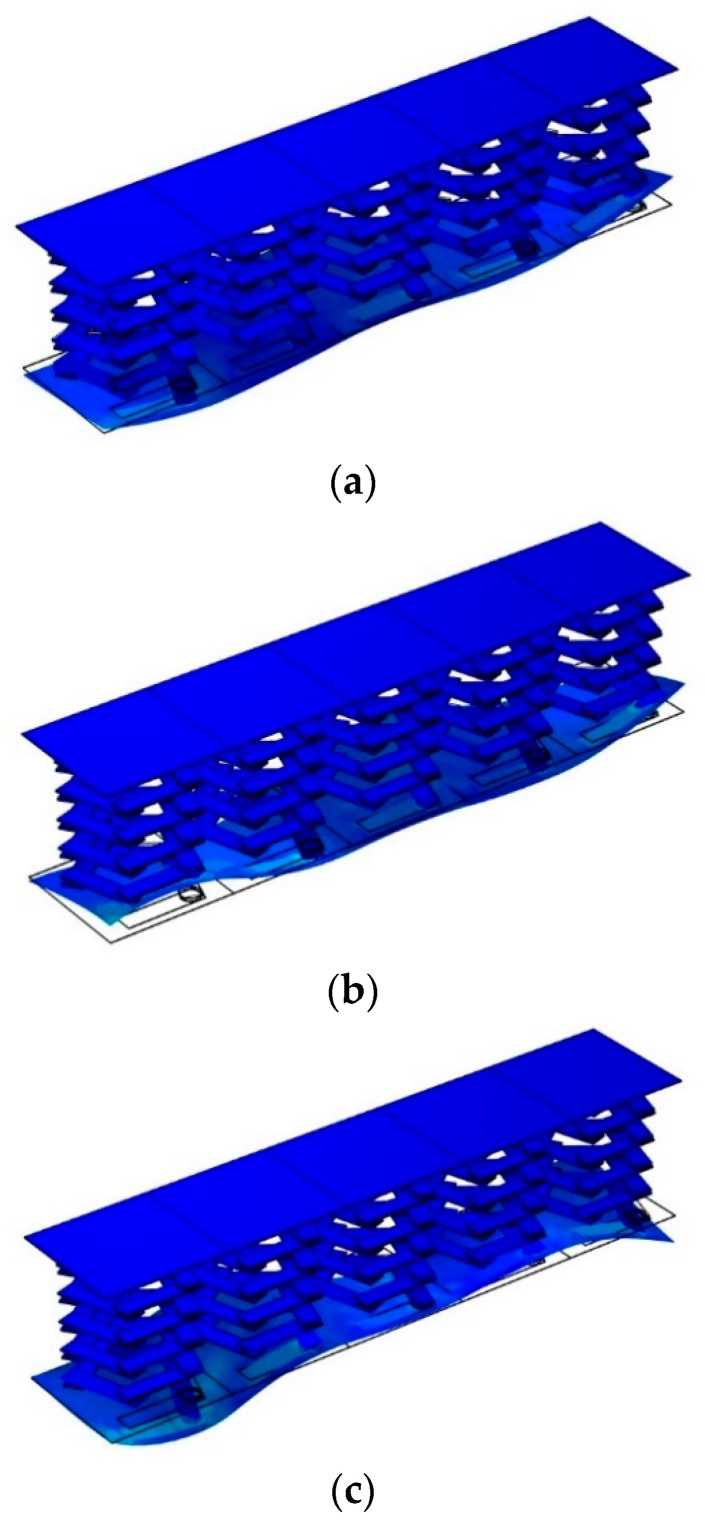
Vibration modes falling in the transmission attenuation frequency band. (**a**) A vibration mode with a frequency of 67 Hz falling in the transmission attenuation frequency band. (**b**) A vibration mode with a frequency of 110 Hz falling in the transmission attenuation frequency band. (**c**) A vibration mode with a frequency of 120 Hz falling in the transmission attenuation frequency band.

From Figure 17, it can be seen that the in vibration mode falling in the non-transmitted damping band, when the lower plate is assigned a displacement excitation in the z-direction, the vibration will not only be transmitted along the lower plate, but the upper plate will also be affected by the directional displacement excitation and will vibrate. This indicates that in the non-transmitted attenuation band, phononic crystals cannot function to block the elastic wave that can be transmitted from the lower plate to the upper plate. As can be seen from the vibration modes in Figure 18, elastic waves whose frequencies fall within the transmission attenuation band can propagate in the lower panel. However, due to the effect of the local resonance pentagonal phononic crystal, the elastic wave is isolated in the phononic crystal, and the elastic wave transmitted in that direction cannot be transmitted from the lower plate to the upper plate, so the upper plate does not vibrate and is in a stable state. The phononic crystal double-layer beam structure effectively suppresses vibration. However, the presence of a cavity portion inside the structure allows the elastic wave generated by the excitation of the lower plate to propagate freely in the acoustic field within the structure. As for the field outside the structure, the vibration of the structure and the noise acoustic waves from the structural vibration can be considered to be well suppressed. This is the reason why a significant vibration attenuation band is present, but there is no significant bandgap.

### 3.4. Influence of Different Geometric Parameters on Bandgap Range and Maximum Transmission Attenuation Value

**Figure 19:**
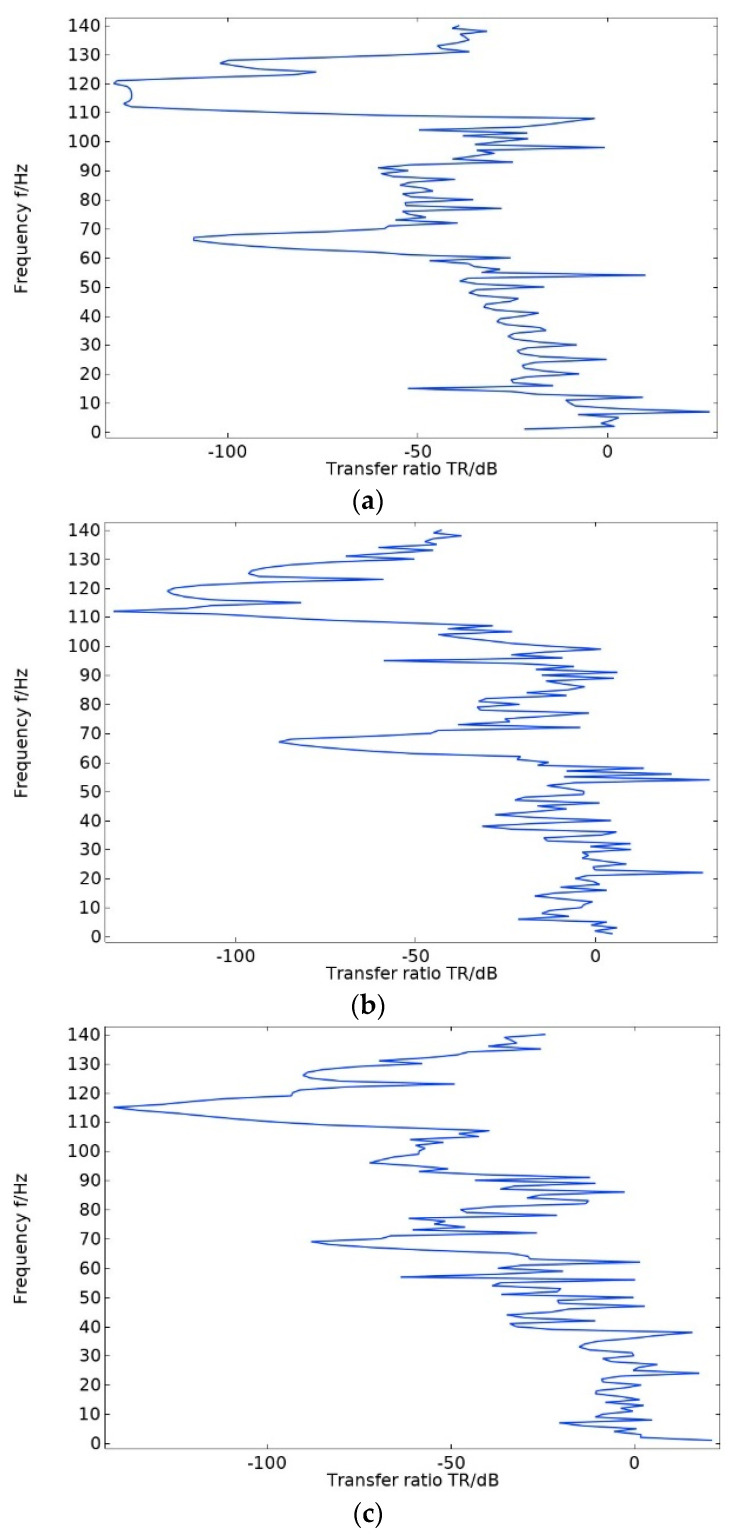
The transmission curve corresponding to the number of different oscillators. (**a**) The transmission curve of a double-layer beam structure with one oscillator. (**b**) The transmission curve of a double-layer beam structure with three oscillators. (**c**) The transmission curve of a double-layer beam structure with five oscillators.

When selecting the number of periodic oscillators as a variable, calculate the transmission curves for oscillators of one, three, and five, respectively. From the transmission curves corresponding to different numbers of oscillators in Figure 19, it can be seen that when the number of oscillators is one, the maximum transmission attenuation value is −129 dB. When the number of oscillators is three, the maximum transmission attenuation value is −134 dB. When the number of oscillators is five, the maximum transmission attenuation value is −142 dB. It can be seen that the maximum transmission attenuation value increases as the number of oscillators increases. In the actual project, the cost and desired effect of vibration and noise reduction can be combined to select the appropriate number of vibrators.

**Figure 20:**
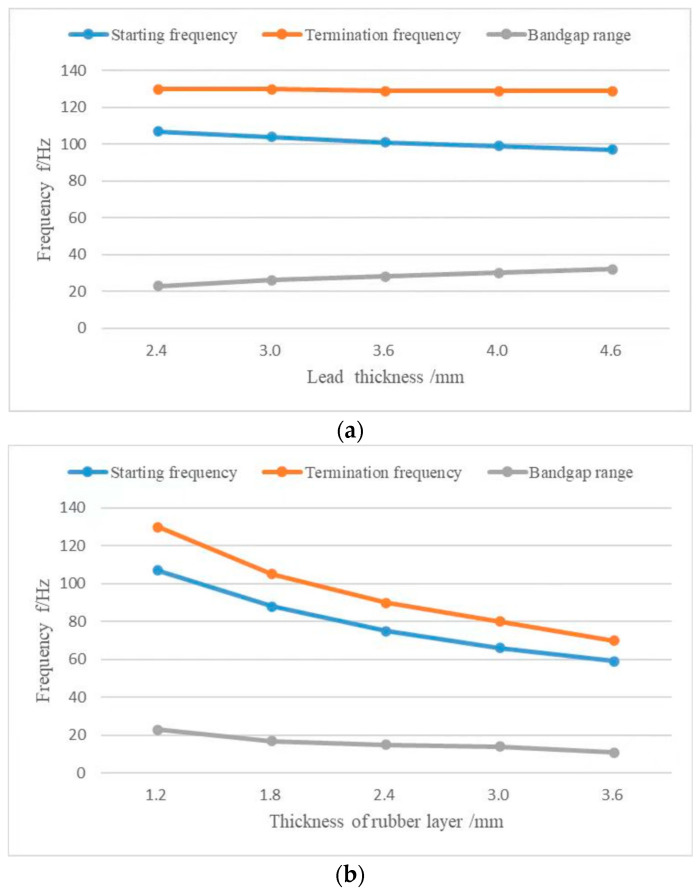

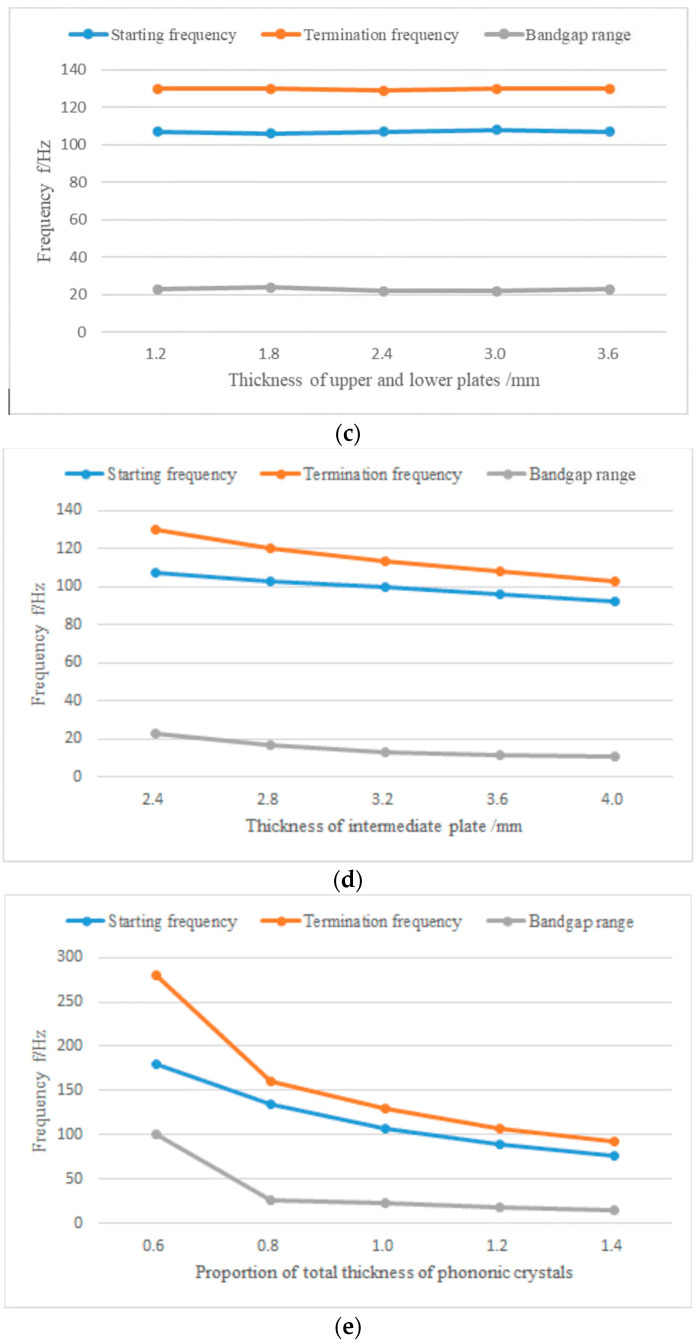

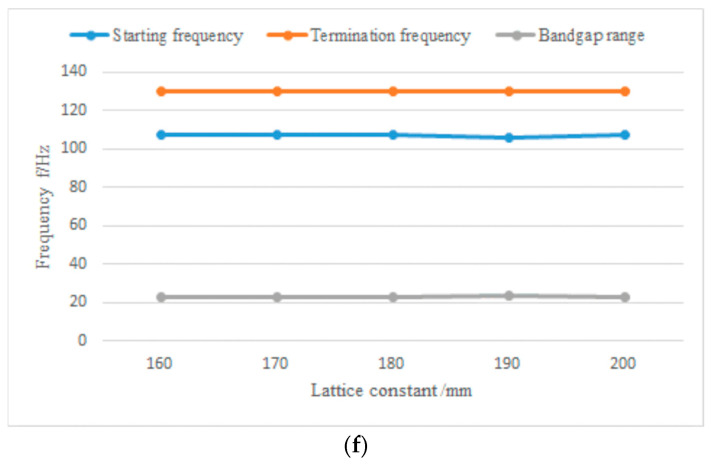
The influence of different parameters on the start and end of the bandgap and the bandgap range. (**a**) Effect of the lead layer’s thickness on the bandgap starting frequency, bandgap ending frequency, and the bandgap range. (**b**) Effect of the rubber layer’s thickness on the bandgap starting frequency, end frequency, and the bandgap range. (**c**) Influence of the upper and lower plates’ thickness on the bandgap starting frequency, end frequency, and the bandgap range. (**d**) Effect of the intermediate plate’s thickness on the bandgap start and end frequencies and the bandgap range. (**e**) Effect of the total thickness ratio of phononic crystals on the bandgap onset and termination frequencies and the bandgap range. (**f**) Effect of the lattice constants on the bandgap start and end frequencies and the bandgap range.

The rubber layer’s thickness $h_{2}$, lead layer’s thickness $h_{3}$, upper and lower plates’ thickness e, intermediate plate’s thickness $h_{4}$, phononic crystal’s thickness and lattice constant a were used as parameter variables to study the effects of their values on the start and end frequency and the bandgap range. From Figure 20a, it can be seen that as the thickness of the lead layer increases, the bandgap onset frequency decreases and the termination frequency remains essentially unchanged, so the bandgap range increases as the onset frequency decreases. As shown in Figure 20b,d, variations in the thickness of the rubber layer and the thickness of the spacer plate have a large effect on the bandgap initiation and termination as well as the bandgap extent. As the thickness of the rubber layer increases, the onset and termination frequencies of the bandgap gradually decrease. However, the corresponding range of the bandgap shows a slight downward trend. As shown in Figure 20c,f, the bandgap onset and termination as well as the bandgap range remain essentially unchanged as the thickness and lattice constant of the top and bottom plates increase. So, the upper and lower plate thicknesses and lattice constants affect the bandgap to a very small extent. As shown in Figure 20e, the total thickness of the phononic crystal has a significant impact on the initiation and termination of the bandgap, as well as its range. As the thickness of the phononic crystal increases, the frequencies at which the bandgap begins and ends decrease rapidly, but the range of the bandgap also decreases.

## 4. Conclusions

In this paper, the energy band structure and transmission curves of the local resonance-type pentagonal phononic crystal beam structure proposed in this paper are calculated using the finite element method and analyzed for the appearance of a bandgap and a transmission-fading band. On this basis, the effects of different geometric parameters on bandgap and transmission attenuation are studied, and the following conclusions are drawn:

Through the analysis of the energy band diagram and the transmission curve of the pentagonal unit oscillator, a bandgap with a width of 33 Hz appeared. Through research, it has been found that the appearance of bandgaps is the result of the coupling of displacement fields corresponding to the vibration modes of pentagonal phononic crystals during local resonance. Therefore, pentagonal phononic crystals have the function of vibration reduction and noise reduction. By comparing the energy band and transmission curves of single-layer and double-layer beams of pentagram phonon crystals, it is concluded that the effect of a single-layer beam on vibration and noise reduction is poor, while that of a double-layer beam is better. By analyzing the effects of different parameters on bandgap and transmission loss, it is concluded that increasing the number of periodic pentagonal phonon crystals is beneficial for increasing transmission loss. Increasing the thickness of lead layer, the thickness of intermediate plate, and the total thickness of phononic crystal can reduce the initial frequency of the bandgap and increase the width of the bandgap. Increasing the thickness of the rubber layer will result in a lower frequency bandgap, but the width of the bandgap will decrease accordingly. Increasing the thickness and lattice constant of the upper and lower plates has little effect on the bandgap. The new pentagonal phononic crystal proposed in this paper emerges with low-frequency bandgaps in the frequency range of 60–70 Hz and 107–130 Hz. Compared to other morphological structures, pentagonal phononic crystals are more competitive in reducing low-frequency vibration and noise, with a bandgap range just below 200 Hz. Low-frequency noise below 200 Hz is usually uncomfortable. The sound produced by an engine and the friction of the tires while driving a car is considered to be low-frequency noise below 200 Hz. The low-frequency noises below 200 Hz produced by aircraft engines and the airflow friction sounds of the fuselage during flight are referred to as operation noises and airflow friction sounds, respectively. The noise of the propeller is also below 200 Hz when a ship is traveling at high speed. Properly installing pentagonal phononic crystal structures on car bodies, fuselages, and ships can effectively reduce the impact of low-frequency noises on people. Therefore, the local resonance-type pentagonal phononic crystal proposed in this paper can be utilized in automotive, aerospace and marine applications.

## Figures and Tables

**Table 1:** Material parameters.

Material	Density/(kg·m^−3^)	Modulus of Elasticity × 10^10^/(N·m^−2^)	Poisson’s Ratio
Steel	7780	21.060	0.300
Lead	11,600	4.080	0.369
Rubber	1300	$1.175\times 10^{-5}$	0.469

**Table 2:** Geometrical parameter.

Parameter	a	e	$h_{1}$	$h_{2}$	$h_{3}$	$h_{4}$	h	l	m
Numerical value	160	1.2	1.2	1.2	2.4	2.4	36	100	24

## Data Availability

Data are contained within the article.
